# Effect of Metal Oxide Nanoparticles on the Breakdown Voltage of Transformer Oil Containing Cellulose Particles

**DOI:** 10.3390/nano15231758

**Published:** 2025-11-24

**Authors:** Tarek S. Negm, Diaa-Eldin A. Mansour, Ahmed A. Hossam-Eldin

**Affiliations:** 1 Department of Electrical Engineering, Faculty of Engineering, Alexandria University, Alexandria 21544, Egypt; tarek.negm@alexu.edu.eg (T.S.N.); a.hossamudn@alexu.edu.eg (A.A.H.-E.); 2 Electrical Power and Machines Engineering Department, Faculty of Engineering, Tanta University, Tanta 31511, Egypt; 3 Electrical Power Engineering Department, Faculty of Engineering, Egypt-Japan University of Science and Technology (E-JUST), New Borg El Arab City, Alexandria 21934, Egypt

**Keywords:** transformer oil, cellulose contamination, nanofluids, impulse breakdown voltage, relative permittivity, dissipation factor

## Abstract

Failures are sometimes attributed to the deterioration of insulating oil, with contamination by cellulose particles. Such contamination lowers the dielectric strength of the oil. This study investigates the effect of cellulose contamination on the impulse breakdown voltage of transformer oil and evaluates the potential of nanofluids as a remediation strategy. A controlled amount of cellulose particles is added and dispersed into mineral oil at a concentration of 0.02 g/L to simulate a contaminated oil sample. Titanium dioxide (TiO_2_) and aluminum oxide (Al_2_O_3_) nanoparticles are then dispersed into the contaminated oil at concentrations of 0.02 and 0.04 g/L. Impulse breakdown voltage is measured under both positive and negative polarities using electrode gaps of 1 mm and 2.5 mm, while dielectric permittivity is also measured to assess polarization effects. The influence of nanoparticle type and concentration is analyzed considering relaxation time and electron scavenging mechanisms. The results show that cellulose contamination markedly reduces dielectric strength, whereas the addition of nanoparticles effectively restores and, in several cases, enhances the insulating properties beyond those of uncontaminated oil.

## 1. Introduction

Liquid dielectrics play a vital role in power systems across both medium and high voltage networks. Among the critical components in these networks, transformers serve as essential parts for stepping voltage levels up or down. Depending on the insulation system employed, transformers are generally categorized into three types: dry-type, gas-insulated, and oil-immersed. Oil-immersed transformers, which are the most widely used, consist of a welded steel tank filled with insulating oil. The oil serves two fundamental functions: it acts as a cooling medium to dissipate heat generated during operation, and it provides the needed electrical insulation between energized components. Compared with other types, oil-immersed transformers are preferred because they can handle higher voltage levels and larger power ratings, offer superior cooling performance, and provide greater operational reliability at relatively lower cost. For these reasons, they dominate in modern electrical power systems.

Different types of insulating liquids are available for use in transformers, including mineral oils, natural esters, synthetic esters, silicone oils, and gas-to-liquid oil [1,2,3,4]. Each of these oils offers distinct advantages and limitations in terms of dielectric strength, thermal stability, biodegradability, and cost. Among these, mineral oil remains the most widely adopted. As a refined petroleum by-product composed of numerous hydrocarbon molecules, it combines favorable dielectric properties with efficient heat transfer capability and cost-effectiveness. These attributes have secured its status as the primary insulating liquid in the majority of power transformers worldwide.

During transformer operation, the insulation system is exposed to a range of electrical, thermal, mechanical, and chemical stresses, either individually or in combination, that gradually degrade its performance [5,6,7]. Electrical stresses caused by electric fields can trigger partial discharges and ionization, weakening the dielectric strength of the oil. Thermal stresses caused by prolonged heating accelerate oxidation and decomposition of both the oil and the solid insulation. Mechanical vibrations and electrodynamic forces arising from load fluctuations or external faults impose structural fatigue. Chemical reactions, including the formation of acids and sludge, further deteriorate the insulating liquid. The interaction of these stresses produces a synergistic effect, sometimes leading to premature aging of the oil–paper insulation system. As degradation progresses, the insulating oil loses its dielectric strength, thereby reducing transformer reliability and significantly increasing the risk of failure if not properly monitored and maintained [8].

In addition to these processes, the transformer core and the insulating paper between windings gradually degrade and decompose during service, releasing contaminants into the oil. These contaminants severely weaken transformer oil by reducing its insulating capability. In [9], metallic particles with a diameter of 150 µm and a weight of 1.0 g are added into one liter of oil, corresponding to a particle concentration on the order of 10^−2^ g/L after uniform circulation. In uncontaminated oil, the breakdown voltage increased monotonically with rising temperature, and the breakdown field ranged between 9.5 kV/mm and 13.4 kV/mm. When metallic particles are added, the breakdown voltage varies with temperature in a non-monotonic way. As the oil temperature increased from 40 °C to 80 °C, the breakdown voltage first increased, reaching a maximum around 70 °C, and then decreased. The peak breakdown field in the contaminated oil is about 6.9 kV/mm, much lower than in pure oil.

Among the various types of impurities, there are cellulose particles originating from the aging and decomposition of insulating paper. In [10], the effect of cellulose particle contamination on the lightning impulse breakdown voltage (LIBV) of mineral oil is systematically investigated under quasi-uniform field conditions. Microcrystalline cellulose particles with an average size of 20 µm are dispersed at concentrations of 0.004, 0.008, and 0.012 wt%, both with and without induced bridge formation. The results demonstrated that cellulose contamination significantly reduced the LIBV, with reductions of up to 27% for non-bridging samples and up to 26.9% when bridging occurred, compared with pure oil. In [11], the breakdown behavior of transformer oil contaminated with cellulose particles is studied under flowing conditions using both AC and DC voltages. Cellulose particles with diameters ranging from 1 to 100 µm are introduced into mineral oil at a mass fraction of 0.003%, and tests are conducted at flow speeds varying from 0 to 0.2 m/s, both with and without voltage preload. The results showed that at low flow velocities (0.01–0.06 m/s), the characteristic breakdown voltage (BDV) decreased compared with static oil due to accelerated cellulose bridging, while at higher velocities (≥0.1 m/s), BDV recovered or even improved as strong flow inhibited bridge formation. Preload conditions consistently led to wider and denser bridges, yielding lower BDV values than non-preload cases, particularly at low speeds.

The experimental results in [12,13] indicate that both the impulse waveform characteristics and the concentration of cellulose particles significantly influence the breakdown voltage of transformer oil. Specifically, an increase in the level of cellulose contamination leads to a consistent reduction in breakdown voltage under a constant impulse wave shape. Likewise, for a fixed contamination level, longer impulse durations result in lower breakdown voltages. The detrimental impact is most pronounced when high levels of cellulose contamination coincide with long-duration impulses, primarily due to enhanced particle mobility within the oil, which facilitates the formation of conductive paths and promotes electrical breakdown.

Recently, researchers have increasingly explored advanced methods to enhance the insulation performance of transformer oils. One promising approach is the use of nanofluids, which are formed by dispersing nanoparticles into transformer oil to improve its dielectric and thermal properties. The concept of nanofluids was first introduced by Choi and Eastman in 1995 [14], primarily aiming to enhance the thermal conductivity of base fluids [15,16,17]. Later, this concept was extended to liquid dielectrics, where preliminary experiments revealed improvements in ac breakdown strength, impulse breakdown voltage, and dielectric losses [18,19,20,21]. Since then, research in this field has expanded rapidly, investigating a wide range of nanoparticles, including metals, metal oxides, carbides, and carbon nanotubes, dispersed in base fluids such as mineral oil, ester oil, and silicone oil [22,23,24,25]. These studies consistently demonstrate that nanofluids can significantly enhance dielectric strength and thermal management.

Different mechanisms have been proposed to explain these improvements. For conductive nanoparticles, the dominant effect is electron scavenging, whereby nanoparticles capture high-energy electrons and convert them into slow-moving charged particles. Their low mobility suppresses space-charge formation at the streamer tip, weakening the local electric field and delaying streamer propagation. This accounts for the enhanced positive breakdown strength observed in nanofluids containing conductive nanoparticles [26]. For semiconductive nanoparticles, the improvement is attributed to the shallow trap effect [27]. Shallow traps capture fast electrons and release them slowly, thereby reducing mobility, delaying streamer development, and extending breakdown time. A third mechanism is related to the interfacial zone formed at the boundary between nanoparticles and the surrounding oil [28]. Due to differences in permittivity and conductivity, this interfacial region exhibits distinct polarization characteristics and can act as an additional barrier against charge transport. The large interfacial area provided by well-dispersed nanoparticles increases charge trapping capacity and enhances local field distortion, thereby strengthening dielectric properties. This interfacial polarization effect, often referred to as the “Maxwell–Wagner–Sillars (MWS) polarization”, contributes significantly to improving breakdown voltage and overall insulation reliability in nanofluids.

The effectiveness of nanofluids also depends on the weight fraction of nanoparticles. Increasing concentration generally enhances dielectric properties up to a certain threshold, beyond which agglomeration and stability issues degrade performance [29]. Thus, selecting the optimal nanoparticle type, size, concentration, and dispersion method remains a critical challenge.

Despite the advances achieved by nanofluids in pure transformer oil, cellulose particles remain one of the most critical contaminants, particularly under impulse voltage stress. To address this challenge, this paper aims to incorporate nanoparticles as a promising pathway to enhance breakdown voltage. Through mechanisms such as electron scavenging, shallow trap effects, and interfacial polarization, nanofluids can counteract the adverse influence of cellulose impurities and help restore dielectric strength. In this work, a controlled amount of cellulose particles is introduced into mineral oil at a concentration of 0.02 g/L to simulate contaminated samples. Titanium dioxide (TiO_2_) and aluminum oxide (Al_2_O_3_) nanoparticles are subsequently dispersed into the contaminated oil at concentrations of 0.02 and 0.04 g/L. This study, therefore, evaluates the effectiveness of these nanoparticle additives in enhancing the dielectric performance of cellulose-contaminated transformer oil, thereby bridging a critical research gap, as most prior studies have examined nanofluids only in uncontaminated oils.

## 2. Materials and Methods

### 2.1. Chemicals Used

Shell Diala S2 ZU-I (from Shell, Cairo, Egypt), a dried uninhibited electrical insulating oil, is selected as the base fluid for preparing the samples. This oil is derived from highly refined mineral oils and is well known for its excellent dielectric properties, high oxidation stability, and efficient heat transfer capability. It complies with the requirements of the IEC 60296 standard [30] and its key specifications are summarized in Table 1.

The objective of this study is to evaluate the effect of dispersing nanoparticles into transformer oil contaminated with cellulose particles. To simulate contamination, cellulose microcrystalline powder with an average particle size of 20 μm, supplied by Sigma-Aldrich (Saint Louis, MO, USA), is added to the base oil, whose main properties are summarized in Table 2.

Following this, two types of metal oxide nanoparticles are dispersed into the contaminated oil to investigate their potential for performance enhancement. Metal oxide nanoparticles are chosen because of their high electronegativity, which enables them to attract fast-moving electrons and delay breakdown processes, as well as their favorable stability characteristics in suspension.

The essential physical properties of the metal oxide nanoparticles employed, namely Al_2_O_3_ and TiO_2_, are summarized in Table 3. Both types of nanoparticles are obtained from Sigma-Aldrich with an average particle size not exceeding 50 nm.

### 2.2. Preparation of Contaminated Oil

The preparation of contaminated oil samples involved dispersing cellulose particles in the base oil at controlled concentrations. Based on the CIGRÉ Technical Brochure [31], transformer oil contamination is categorized into five levels: Nil, Low, Normal, Marginal, and High. Previous studies [12,13,32,33,34] have shown that cellulose contamination can significantly influence the electrical and thermal properties of mineral oils. Among the different quantification methods, the weight-per-volume ratio is the most commonly used to represent cellulose concentration relative to the base oil. Considering that 0.01 g/L represents an average contamination level in service transformers and 0.05 g/L a marginal level [31,34], a cellulose concentration of 0.02 g/L is selected for this work to simulate a practical and experimentally reproducible intermediate contamination level. Even though cellulose powder particles have been used by default by CIGRÉ and others, this does not mean that they are fully representative of cellulose fibers actually present in the oil of transformers in terms of BV resistance.

### 2.3. Preparation of Nanofluids

The preparation of nanofluids requires careful control to ensure stable and homogeneous suspensions, as nanoparticle agglomeration can degrade their nanoscale properties [35]. Among the two common preparation techniques, the single-step and two-step methods, the latter is adopted in this study due to its practicality and reproducibility. In the two-step method, nanoparticles are first synthesized or obtained commercially and then dispersed into the base fluid under controlled physical treatments.

**Figure 1:**
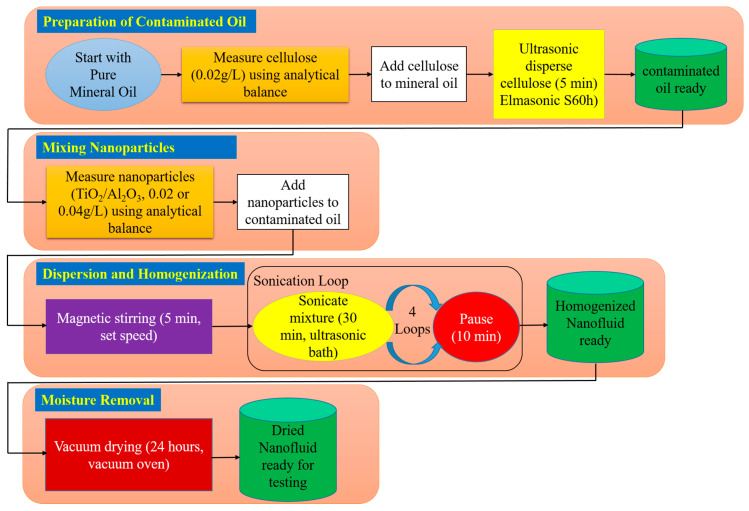
Flowchart of the preparation process of contaminated oil-based nanofluids.

As shown in Figure 1, preparation began by forming contaminated oil through the dispersion of 0.02 g/L cellulose microcrystalline powder, whose weight is measured using a five-digit analytical balance (from KERN, Balingen, Germany), into pure mineral oil. The mixture is ultrasonically treated in an Elmasonic S60h bath (from Elma Schmidbauer, Singen, Germany) for five minutes to achieve uniform distribution. Subsequently, the required amount of Al_2_O_3_ or TiO_2_ nanoparticles is weighed using the same analytical balance and introduced into the pre-contaminated oil. The nanofluid is first magnetically stirred (stirrer from IKA-Werke, Staufen, Germany) for five minutes, followed by ultrasonic homogenization by Elmasonic S60h for a total of two hours (four 30 min cycles with 10 min intervals) to minimize agglomeration and ensure uniform dispersion.

Finally, owing to the hygroscopic nature of the process and the likelihood of ambient moisture absorption during sonication, a factor known to impair dielectric breakdown strength, the prepared nanofluids are vacuum-dried for 24 h in a vacuum oven (from BINDER, Tuttlingen, Germany). This final step ensured the removal of excess moisture, producing dried nanofluid samples suitable for testing.

### 2.4. Nanofluid Stability Evaluation

Before deciding the suitable weight fraction of the added nanoparticles, the stability of the prepared nanofluids is first evaluated quantitatively by measuring their optical absorbance using UV-Vis spectroscopy (from Malvern Panalytical, Worcestershire, UK). Absorbance measurements are performed over a range of nanoparticle weight fractions from 0.001 g/L to 0.1 g/L for Al_2_O_3_-based nanofluids. The measured absorbance values are compared with theoretical values predicted by the Beer–Lambert law, and the percentage deviation between the two is calculated to assess the degree of nanoparticle dispersion stability.

**Figure 2:**
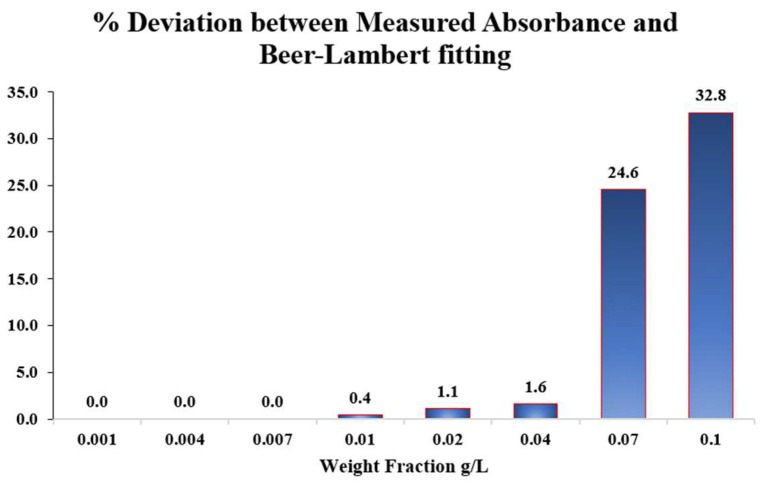
Percentage deviation between the measured absorbance value of Alumina nanofluid and the theoretical value based on the linear Beer-Lambert relation.

For the samples containing nanoparticle concentrations of 0.001, 0.004, and 0.007 g/L, the measured absorbance values exhibit a linear trend, consistent with the Beer-Lambert law. The slope of this linear regression was determined and used to establish the theoretical Beer–Lambert relationship for the Alumina nanofluid. Subsequently, higher weight concentrations of 0.01, 0.02, and 0.04 g/L were prepared, and their absorbance values were experimentally measured and compared with the corresponding theoretical values. The percentage deviations between the measured and theoretical absorbance data were found to be 0.4%, 1.1%, and 1.6% at 0.01, 0.02, and 0.04 g/L, respectively, confirming that the suspension remained stable and well-dispersed at these concentrations. In contrast, samples prepared at higher concentrations of 0.07 and 0.1 g/L exhibited considerably larger deviations of 24.6% and 32.8%, respectively. These significant discrepancies indicate nanoparticle agglomeration, which effectively reduces the available surface area and hence alters the optical response of the suspension. Figure 2 shows the percentage deviation between the measured absorbance and the theoretical Beer–Lambert prediction across the investigated Alumina nanoparticle weight fractions.

Based on these findings, nanoparticle weight fractions of 0.02 and 0.04 g/L are selected for subsequent dielectric testing, as they exhibited optimal dispersion and minimal deviation from the Beer–Lambert relation, confirming stable suspension behavior. For further verification, the absorbance of these selected samples is re-measured 24 h after preparation and found to be identical to the initial readings, confirming the temporal stability of the nanofluids. The same procedure is repeated for Titania nanofluid samples, yielding the same results.

**Figure 3:**
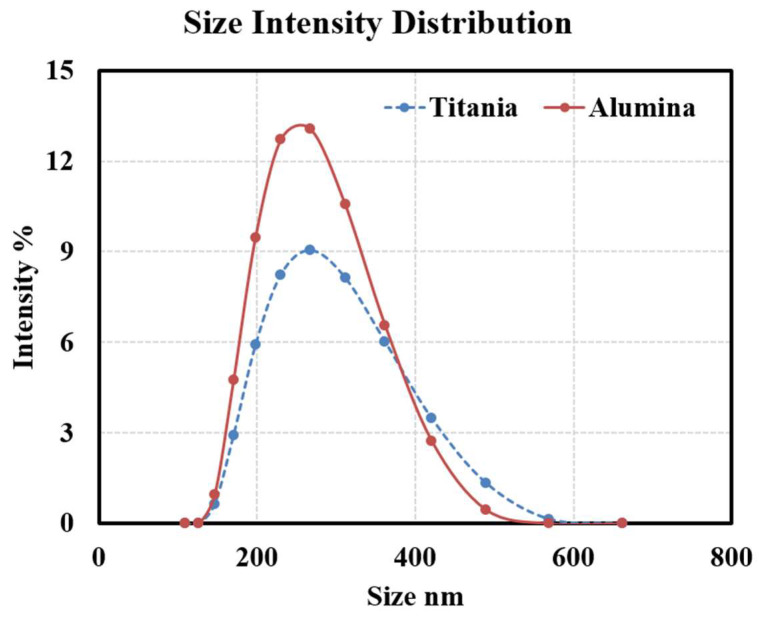
Dynamic Light Scattering (DLS) analysis of nanoparticles used within the oil.

Moreover, Figure 3 shows the Dynamic Light Scattering (DLS) results for both Al_2_O_3_ and TiO_2_ oil-based nanofluids, including the nanoparticle distribution and average particle size within the oil. The distribution shows that both types of nanoparticles have small diameters after being added to base oil, indicating good dispersion and a homogeneous nanofluid. Despite all these precautions to ensure the stability of nanoparticles in oil, only long-term (months or years) studies of such particles in transformers in service will tell if they will re-agglomerate or not into larger ones over time.

### 2.5. Impulse Test Setup

The dielectric strength of the prepared samples is evaluated using an impulse breakdown test, which provides more representative insights than conventional AC breakdown testing. Preliminary AC tests revealed negligible differences between the breakdown voltages of cellulose-contaminated and pure oils, consistent with previous findings [36,37] that low levels of cellulose contamination have minimal influence on AC dielectric strength.

The limited effectiveness of AC testing arises from the forces acting on cellulose particles suspended in the oil. Under a uniform AC field, the dielectrophoretic and Coulomb forces cause particles to oscillate near the electrodes without forming a conductive bridge, preventing consistent breakdown initiation. Consequently, the influence of contaminant particles or nanoparticle additives cannot be clearly identified in this configuration.

**Figure 4:**
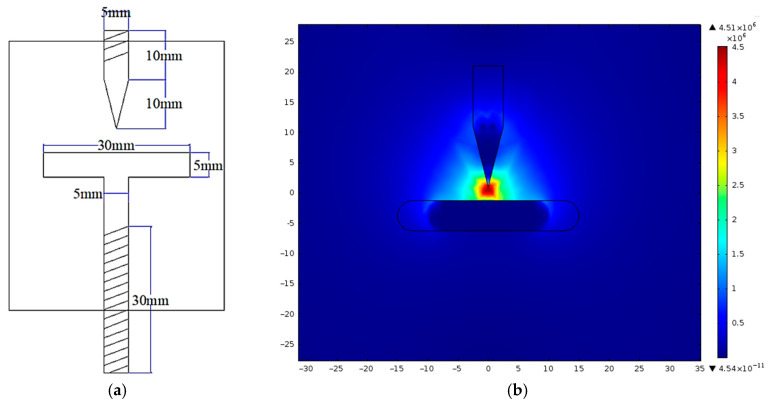
Needle-to-plate electrodes Test Cell: (**a**) dimensions of electrodes; (**b**) electric field distribution.

To overcome these limitations, impulse voltage testing is employed. This method introduces a non-uniform electric field, allowing a more accurate assessment of how cellulose contamination deteriorates dielectric strength and how nanoparticle addition reduces this effect. A custom needle-to-plate electrode configuration is designed and fabricated for this purpose, as illustrated in Figure 4a. The corresponding electric field distribution, simulated using FEMM 4.2 software, is presented in Figure 4b. This configuration replicates the non-uniform fields typically present near sharp edges and structural irregularities in transformer components, making it a realistic and sensitive setup for studying breakdown phenomena.

Therefore, evaluating the dielectric behavior of the oil under these conditions provides a more accurate representation of operational stress. The impulse breakdown tests are conducted in accordance with IEC 60897 standard procedures [38].

**Figure 5:**
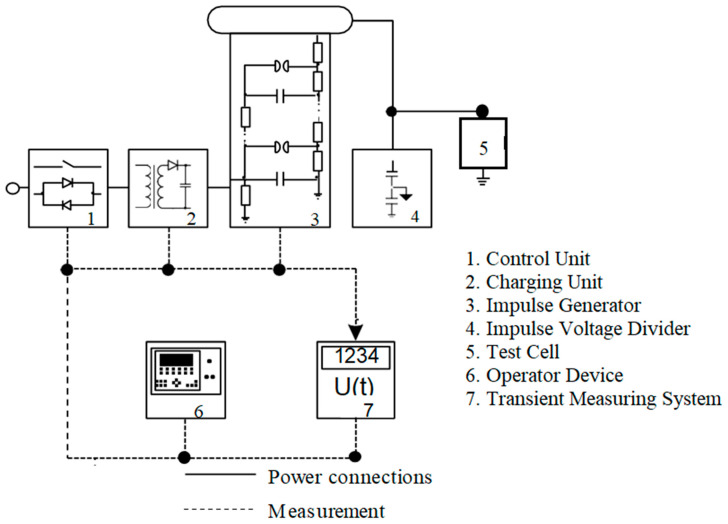
Experimental setup for the impulse test.

The experimental setup used to generate high-voltage electrical impulses is illustrated in Figure 5 (from High Volt, Dresden, Germany). The impulse generator employed is a single-stage Marx circuit, capable of producing a total charging voltage of 100 kV and delivering a stored energy of 2.5 ± 5% kJ. Charging is accomplished through a dedicated charging unit, which is interfaced with a control unit connected to the main power supply.

A 500 ± 5% nF load capacitor is utilized as a damped capacitive voltage divider, suitable for both lightning and switching impulse applications. Voltage measurements are captured using a transient recorder integrated into the system. The entire test circuit is operated via a user interface device that communicates with the control unit via fiber-optic cables. Additionally, trigger signals for the impulse generator are transmitted optically to ensure electrical isolation and minimize electromagnetic interference. This configuration ensures stable, interference-free operation of the testing system.

### 2.6. Dielectric Spectroscopy Test

Relative permittivity and dissipation factor are crucial parameters for electrical engineers, as they determine the suitability of dielectric materials for high-voltage applications. Relative permittivity quantifies the extent of polarization in both the base oil and the nanofluid samples.

Permittivity and dissipation factor measurements are performed using a precision LCR meter Keysight E4980A (by Keysight Technologies, Santa Rosa, CA, USA), which offers a basic accuracy of 0.05% and ensures excellent measurement repeatability across both low- and high-impedance ranges. The measured values reflect the interaction between the applied external electric field and the electric dipole moments within the sample. Relative permittivity is evaluated as a function of frequency over a broad range of 20 Hz to 2 MHz. This wide frequency spectrum is particularly important, as the dielectric response is governed by the relaxation time, which varies with the applied frequency. It should be emphasized that polarization in dielectric materials does not occur instantaneously upon the application of an external field, but instead exhibits a frequency-dependent delay due to the underlying relaxation mechanisms.

**Figure 6:**
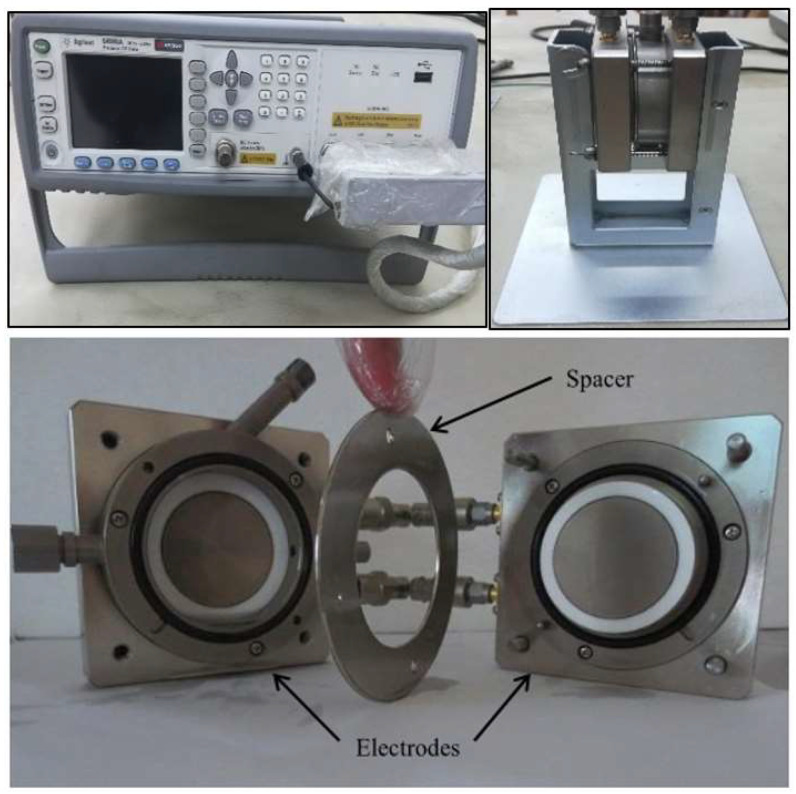
Setup for the Dielectric Spectroscopy Test (LCR meter).

The oil sample is placed in the LCR meter test cell shown in Figure 6, which consists of two parallel electrodes with a diameter of 38 mm, separated by a distance of 0.3 mm. A variable frequency voltage is applied to the sample, and the magnitude and phase of the resulting current flowing through the sample are subsequently measured.

## 3. Results

After performing impulse tests, there are four groups of results; the first shows the effect of adding cellulose particles as a contaminant to the transformer oil to determine which percentages of added cellulose have the greatest effect on deteriorating the dielectric characteristics. The second and third groups are concerned with the effects of adding Titania and Alumina nanoparticles, respectively, to restore the dielectric properties of the contaminated oil. The fourth and last group of results shows the dielectric measurements from the LCR meter represented in the relative permittivity and dissipation factor.

### 3.1. Cellulose-Induced Deterioration in Transformer Oil

**Figure 7:**
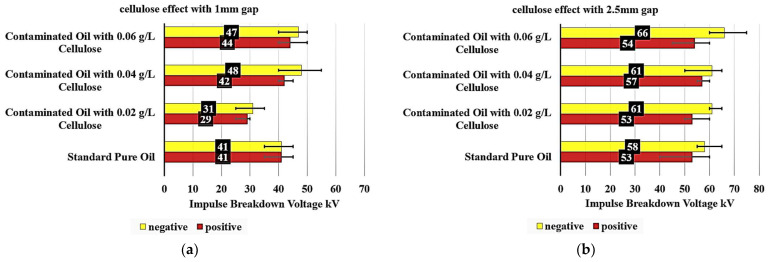
Cellulose-induced deterioration in pure oil: (**a**) with 1 mm electrode gap; (**b**) with 2.5 mm electrode gap.

An examination of Figure 7a provides detailed insights into how cellulose contamination affects the breakdown voltage of insulating oil. The reference sample, composed of standard pure oil, demonstrated a breakdown voltage of 41 kV at a 1 mm gap, serving as the baseline for comparison. In the case of oil contaminated with 0.02 g/L of cellulose, a noticeable reduction in breakdown voltage is observed. Under positive polarity, the breakdown voltage decreased by approximately 29.3%, reaching 29 kV. Under negative polarity, the reduction is slightly less pronounced, at 24.4%, resulting in a breakdown voltage of 31 kV. These results indicate that even low levels of cellulose contamination can significantly degrade the dielectric strength of the insulating oil.

When the cellulose concentration is increased to 0.04 g/L, the breakdown voltage shows a different trend. Under positive polarity, a slight increase of 2.4% is observed, reaching 42 kV. In contrast, the negative polarity shows a more significant increase of 17.1%, with the breakdown voltage rising to 48 kV.

These results suggest that the relationship between cellulose concentration and dielectric strength is not straightforward and may involve complex interactions.

At the highest tested cellulose concentration of 0.06 g/L, the positive polarity breakdown voltage increases further by 7.3%, reaching 44 kV compared to the standard oil. Notably, the negative polarity also shows a strong response, with the breakdown voltage reaching 47 kV, an increase of 14.6% over the pure oil, though this is slightly lower than the peak value observed at 0.04 g/L. This behavior indicates a potential saturation effect or a shift in the particle-field interactions at higher contamination levels.

When analyzing Figure 7b, the data reveal nuanced insights into the impact of cellulose contamination on the dielectric properties of insulating oils.

The baseline “Standard Pure Oil” sample exhibits a positive polarity breakdown voltage of 53 kV and a negative polarity breakdown voltage of 58 kV, establishing the reference points for comparison. Upon introducing cellulose contamination, the positive and negative polarity breakdown voltages exhibit divergent responses.

At the 0.02 g/L cellulose concentration, the positive polarity breakdown voltage remains unchanged at 53 kV, while the negative polarity experiences a modest 5.2% increase to 61 kV. This suggests that low levels of cellulose contamination have a more pronounced impact on the negative polarity breakdown characteristics. Increasing the cellulose content to 0.04 g/L, the positive polarity breakdown voltage increases by 7.5% to 57 kV, whereas the negative polarity maintains the same 61 kV value as the 0.02 g/L sample. This indicates a non-linear relationship between cellulose concentration and the dielectric performance. At the highest tested cellulose level of 0.06 g/L, the positive polarity breakdown voltage increases by 1.9% to 54 kV, compared to the standard oil. The negative polarity breakdown voltage demonstrates the most substantial improvement, increasing by 13.8% to 66 kV.

The previous results have demonstrated that the oil sample contaminated with 0.02 g/L of cellulose particles exhibits the lowest impulse breakdown voltage. This suggests that this specific concentration of cellulose contamination has the most significant effect on the dielectric properties of the oil.

Consequently, this contaminated oil sample has been selected for further investigation. The objective is to study the effect of adding nanoparticles to the contaminated oil and assess how the nanoparticle addition influences the dielectric characteristics of the oil. This research aims to provide insights into the potential of using nanoparticle additives to decrease the detrimental impact of cellulose contamination on the dielectric performance of insulating oil.

### 3.2. Mitigation Effect of TiO_2_ (Titania)

**Figure 8:**
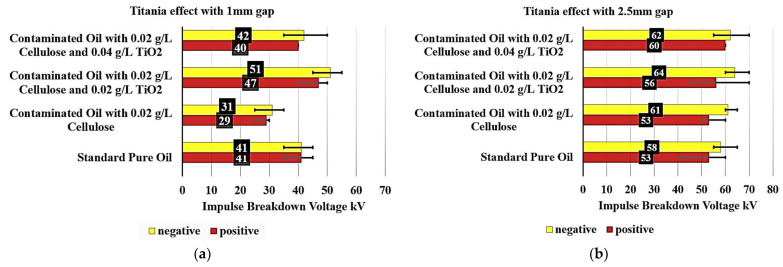
Titania Mitigation of Cellulose-induced deterioration in contaminated oil: (**a**) with 1 mm electrode gap; (**b**) with 2.5 mm electrode gap.

Figure 8a examines the Titania effect on the breakdown voltage of various contaminated oil samples with 0.02 g/L cellulose particles with a 1 mm gap. The standard pure oil exhibited a consistent breakdown voltage of 41 kV under both negative and positive polarity conditions, serving as a benchmark.

When analyzing the contaminated oil samples, notable differences are observed. The contaminated oil with 0.02 g/L cellulose showed a significant decrease in breakdown voltage, reaching 31 kV under negative polarity (a 24.4% decrease) and 29 kV under positive polarity (a 29.3% decrease). This significant reduction in breakdown voltage can be attributed to the presence of cellulose contaminants in the oil.

Interestingly, the addition of TiO_2_ to the contaminated oil samples had a positive impact on the breakdown voltage performance. The contaminated oil with 0.02 g/L cellulose and 0.02 g/L of TiO_2_ exhibited a breakdown voltage of 51 kV under negative polarity (24.39% increase) and 47 kV under positive polarity (a 14.63% increase), surpassing the standard pure oil. This suggests that the inclusion of a small amount of TiO_2_ can effectively reduce the detrimental effects of cellulose contamination on the oil’s insulation properties.

Furthermore, the contaminated oil with 0.02 g/L cellulose and 0.04 g/L of TiO_2_ also showed improved performance, with a breakdown voltage of 42 kV under negative polarity (a 2.44% increase) and 40 kV under positive polarity (a 2.44% decrease). However, it did not reach the levels of the 0.02 g/L TiO_2_ sample, but it can be said that the contaminated oil became as good dielectric as the pure standard oil without contaminants.

On the other hand, Figure 8b examines the effect of Titania on the breakdown voltage of various contaminated oil samples with 0.02 g/L cellulose particles, but with a 2.5 mm gap. The standard pure oil exhibited a breakdown voltage of 58 kV under negative polarity and 53 kV under positive polarity, serving as a benchmark.

When analyzing the contaminated oil samples, some notable observations are made. The contaminated oil with 0.02 g/L cellulose displayed a breakdown voltage of 61 kV under negative polarity, representing a 5.17% increase compared to the standard pure oil. Under positive polarity, the breakdown voltage remained unchanged at 53 kV.

Interestingly, the addition of TiO_2_ to the contaminated oil samples had a positive impact on the breakdown voltage performance. The contaminated oil with 0.02 g/L cellulose and 0.02 TiO_2_ exhibited a breakdown voltage of 64 kV under negative polarity, a 10.34% increase, and 56 kV under positive polarity, a 5.66% increase, surpassing the standard pure oil.

Furthermore, the contaminated oil with 0.02 g/L cellulose and 0.04 g/L TiO_2_ also showed improved performance, with a breakdown voltage of 62 kV under negative polarity, representing a 6.90% increase, and 60 kV under positive polarity, representing a 13.21% increase. While this sample did not reach the levels of the 0.02 TiO_2_ sample, it still demonstrated a 6.90% increase under negative polarity and a 13.21% increase under positive polarity compared to the standard pure oil.

These findings highlight the importance of understanding the impact of contaminants, such as cellulose, on the electrical properties of insulating oils, as well as the potential benefits of incorporating appropriate additives, like TiO_2_, to enhance the oil’s breakdown voltage and overall insulation performance.

### 3.3. Mitigation Effect of Al_2_O_3_ (Alumina)

**Figure 9:**
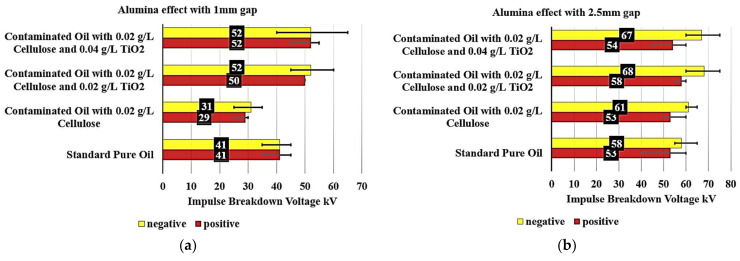
Alumina Mitigation of Cellulose-induced deterioration in contaminated oil: (**a**) with 1 mm electrode gap; (**b**) with 2.5 mm electrode gap.

Figure 9a shows that the addition of 0.02 g/L of Al_2_O_3_ to the contaminated oil with cellulose results in a remarkable improvement in the insulating properties. The breakdown voltage increased by 21.95% to 50 kV for the positive polarity and by 26.83% to 52 kV for the negative polarity. This finding suggests that incorporating Al_2_O_3_ can effectively mitigate the detrimental effects of cellulose contamination, restoring insulating performance to levels comparable to or even surpassing those of standard pure oil. Furthermore, when the Al_2_O_3_ concentration in the contaminated oil is increased to 0.04 g/L, the breakdown voltage remains stable at 52 kV under both polarities. This indicates a saturation point at which additional Al_2_O_3_ provides no further improvement in the insulating properties, potentially informing the optimal dosage for practical applications.

On the other hand, Figure 9b examines the Alumina effect on the breakdown voltage of various contaminated oil samples containing 0.02 g/L cellulose particles, with a 2.5 mm gap. The baseline standard pure oil exhibited a breakdown voltage of 53 kV for positive polarity and 58 kV for negative polarity, establishing the reference performance.

When the oil is contaminated with 0.02 g/L of cellulose, the positive polarity breakdown voltage remained unchanged at 53 kV, while the negative polarity increased slightly by 5.17% to 61 kV. This suggests that the presence of 0.02 g/L cellulose contamination had a minimal impact on the insulating properties, particularly for the negative polarity. The addition of 0.02 g/L Al_2_O_3_ to the contaminated oil with 0.02 g/L cellulose resulted in a significant improvement in the breakdown voltage. For the positive polarity, the voltage increased by 9.43% to 58 kV, while for the negative polarity, it increased by 17.24% to 68 kV. This indicates that the incorporation of Al_2_O_3_ can effectively reduce the detrimental effects of cellulose contamination.

Further increasing the Al_2_O_3_ concentration to 0.04 g/L in the contaminated oil maintained the improved breakdown voltage performance. The positive polarity voltage increased slightly by 1.89% to 54 kV, while the negative polarity remained high at 67 kV, a 15.52% increase over the standard pure oil. This observation suggests a saturation point at which additional Al_2_O_3_ does not provide a substantial further enhancement in the insulating properties.

### 3.4. Dielectric Spectroscopy Test Results

For the same groups of samples under investigation, the LCR meter is used to measure the relative permittivity and dissipation factor, which are important parameters for assessing the dielectric strength of the insulator. Relative permittivity represents the measurement of the amount of polarization in both base oil and Nanofluid samples. On the other hand, the dissipation factor indicates the amount of losses in the insulation, commonly called tan δ.

**Figure 10:**
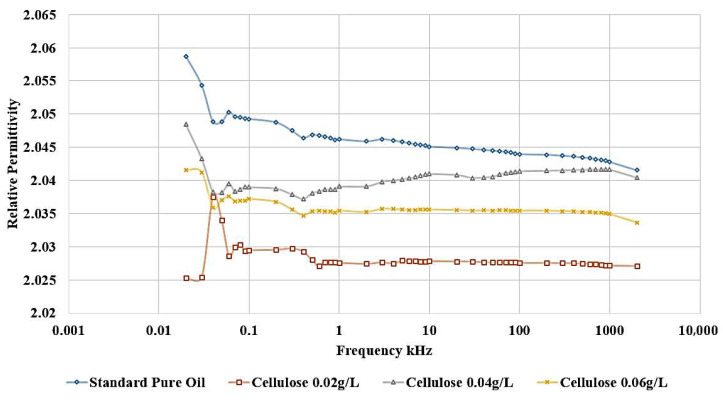
Cellulose effect on the relative permittivity of oil.

**Figure 11:**
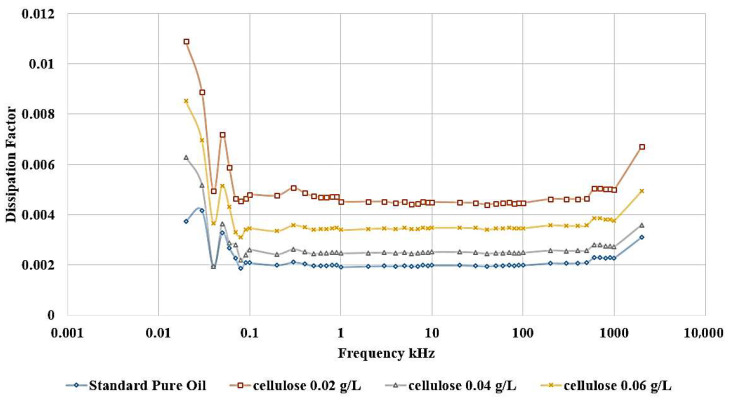
Cellulose effect on Dissipation Factor of oil.

The influence of incorporating cellulose particles into transformer oil at varying weight ratios is depicted in Figure 10 and Figure 11.

The results demonstrate that the introduction of cellulose particles as a contaminant to the oil led to a decrease in the relative permittivity, as shown in Figure 10, which corresponds to a reduction in dielectric strength. The most detrimental effect is observed when adding 0.02 g/L of cellulose, and this contaminated oil sample is subsequently utilized to investigate the impact of incorporating nanoparticles as a means of healing the dielectric liquid. In addition, Figure 11 shows the same effect, as shown, the highest dissipation factor is related to the contaminated sample with 0.02 g/L cellulose.

**Figure 12:**
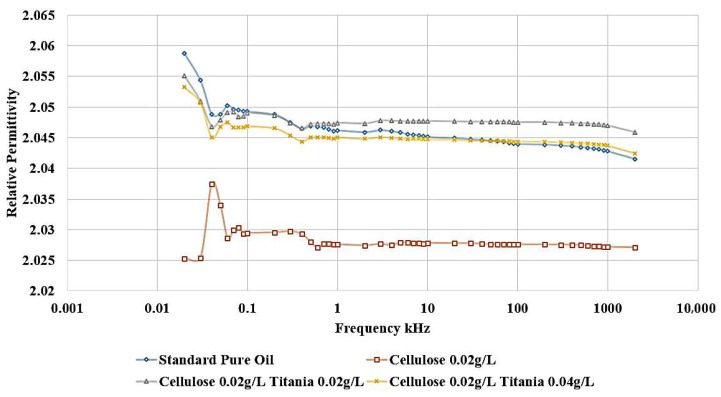
Titania effect on the relative permittivity of contaminated oil with 0.02 g/L of cellulose particles.

**Figure 13:**
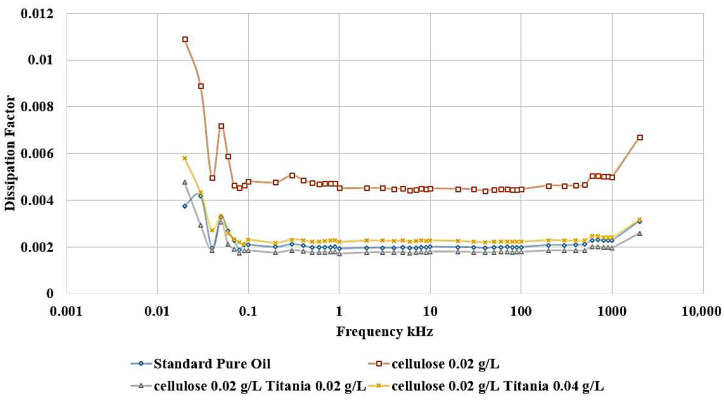
Titania effect on the Dissipation Factor of contaminated oil with 0.02 g/L of cellulose particles.

Adding Titania nanoparticles to the contaminated oil led to a restoration of the relative permittivity to its original value observed in the standard, uncontaminated oil, as shown in Figure 12. Specifically, the addition of 0.04 g/L of Titania nanoparticles is able to recover the relative permittivity to the level of the pure, uncontaminated oil. Furthermore, when 0.02 g/L of Titania nanoparticles are added, the relative permittivity is even observed to be slightly higher than that of the standard, pure oil. The same improvement is observed in the dissipation factor, as shown in Figure 13. After adding nanoparticles, the dissipation factor decreases again to values roughly equal to the standard pure oil value.

These results demonstrate the significant efficiency of Titania nanoparticles in restoring the dielectric characteristics of the oil, even when it has been contaminated by the presence of cellulose particles. The ability of the Titania nanoparticles to decrease the deleterious effects of cellulose contamination highlights their potential as a functional additive for the enhancement of dielectric liquid performance.

**Figure 14:**
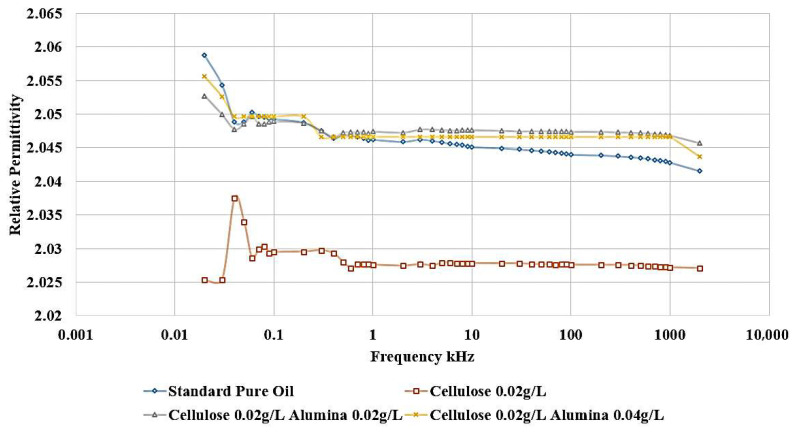
Alumina effect on the relative permittivity of contaminated oil with 0.02 g/L of cellulose particles.

**Figure 15:**
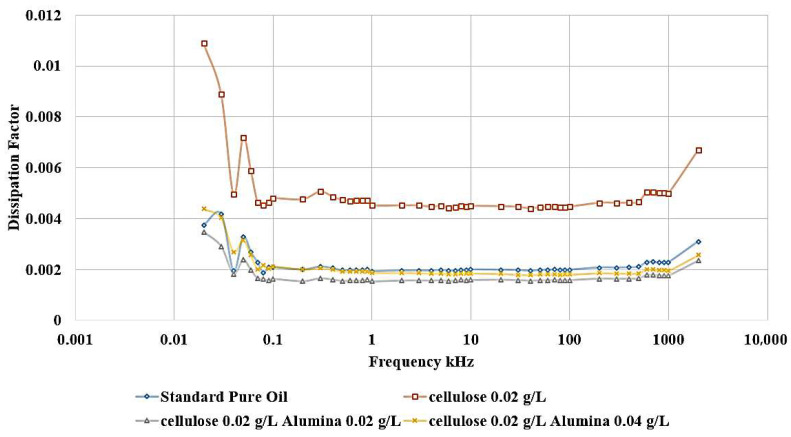
Alumina effect on the Dissipation Factor of contaminated oil with 0.02 g/L of cellulose particles.

Figure 14 shows that the incorporation of Al_2_O_3_ nanoparticles into the contaminated dielectric liquid exhibited a similar effect to that observed with titanium dioxide (TiO_2_) nanoparticles. The addition of 0.04 g/L or 0.02 g/L of Alumina nanoparticles is able to restore the relative permittivity of the liquid to a value slightly higher than that of the standard, uncontaminated pure oil. In addition, a similar enhancement is noticed in the dissipation factor, as shown in Figure 15. After adding nanoparticles, the dissipation factor decreases again to values around the standard pure oil value. These findings demonstrate the effect of Alumina nanoparticles in mitigating the detrimental effects of cellulose contamination on the dielectric properties of the liquid.

The ability of both Titania and Alumina nanoparticles to enhance the relative permittivity and dissipation factor beyond the level of the uncontaminated oil highlights their potential as functional additives for the optimization of dielectric liquid performance.

### 3.5. Summary of Results

**Figure 16:**
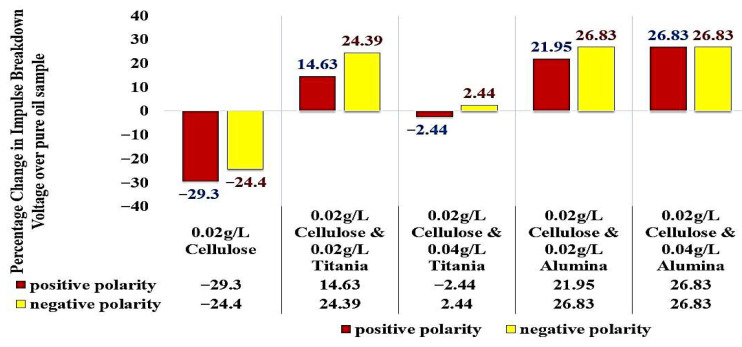
Summary of percentage change in impulse breakdown voltage over pure oil sample (1 mm gap between electrodes).

**Figure 17:**
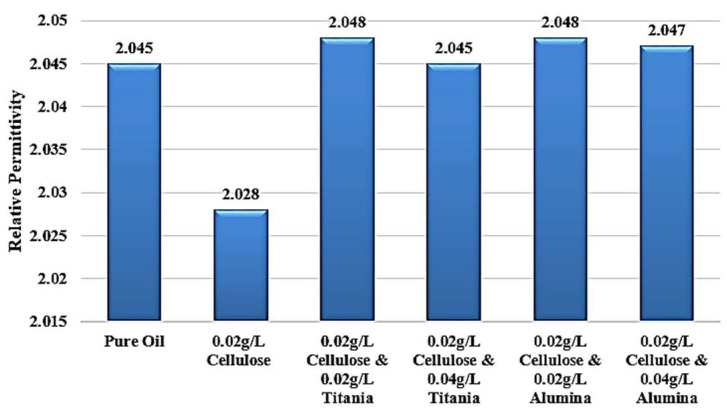
Summary of the average relative permittivity of the samples under investigation.

**Figure 18:**
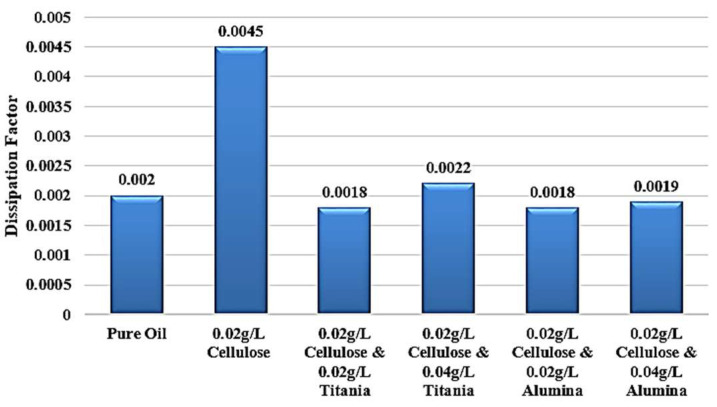
Summary of the average dissipation factor of the samples under investigation.

Considering the standard pure oil as the baseline, Figure 16 shows the percentage change in LIBV upon adding cellulose contaminants and upon adding Titania and Alumina as Nano additives. In addition, relative permittivity and dissipation factor results are summarized in Figure 17 and Figure 18, showing the contribution of adding metal oxide nanoparticles in restoring the dielectric properties of transformer oil after being contaminated by cellulose particles.

## 4. Discussion

There are three main theories that can explain the obtained results: the Moisture Binding Theory, the Electron Scavenging Theory, and the Electronegativity Theory.

The moisture-binding mechanism states that the main influence on the breakdown voltage of liquid dielectric comes from the moisture content [39]. Due to the hydrophilic nature of nanoparticles [40], they bind moisture content on their surfaces. The cellulose particles, which are the main contaminant, are hydrophilic and include most of the moisture content inside the transformer oil. Consequently, nanoparticles, which are also hydrophilic, attract the moisture held within the cellulose particles, effectively binding and neutralizing its detrimental influence on streamer initiation and propagation. This interaction reduces the role of cellulose as a moisture carrier, thereby improving the dielectric strength of the nanofluid.

On the other hand, the Electron Scavenging mechanism was found by George Hwang [26] in 2010. It is concerned with short-time breakdown tests, so it can be considered a good theory to explain impulse breakdown test results. It states that adding nanoparticles to the liquid dielectric slows down the propagation of streamers, resulting in an increase in dielectric strength. This enhancement comes from the polarization process of nanoparticles when applying an electric field. The enhancement in dielectric properties relates to the time required to build up a polarization. This time can be roughly estimated to be the same as the relaxation time of the nanoparticles, which is the time required to return the dipoles to their random distribution after releasing the source of electric field. This theory can be summarized as follows: when an electric field is applied to the nanofluid from the positive to the ground electrode, nanoparticles are aligned in the opposite direction to the electric field, with positive surface charge facing the ground electrode and negative surface charge facing the positive electrode. When a free electron moves from ground to the positive electrode in the opposite direction of the electric field, it faces the positive surface charge of the nanoparticle and becomes stuck to it. During scavenging, more and more electrons are added to the positively charged part of the nanoparticle, and the positive surface area decreases while the negative surface area increases until the nanoparticle becomes completely negatively charged. This can explain how the presence of nanoparticles slows down the streamer as it converts the fast free electrons into negatively charged particles, which are much slower than free electrons. This process can explain the enhancement of dielectric properties after adding the nanoparticles, even if cellulose particles exist. The main factor here is the relaxation time of the nanoparticles. If this time is small, then the particles will be polarized quickly, and the streamer will be delayed, leading to an increase in the breakdown voltage. On the other hand, if this relaxation time is long, the streamer will propagate before the polarization process, leading to a decrease in the breakdown voltage.

Relaxation time of nanofluid can be calculated by the following equation [26]:

(1) $$T=\frac{2\varepsilon_{1}+\varepsilon_{2}}{2\sigma_{1}+\sigma_{2}}$$

where $\varepsilon_{1}$, $\sigma_{1}$ are the permittivity (F/m) and conductivity (S/m) of transformer oil and $\varepsilon_{2}$, $\sigma_{2}$ are the permittivity and conductivity of nanoparticles.

From Table 4 and Equation (1), the relaxation time constants of Alumina-based and Titania-based nanofluids are 41.3 s and 438.9 s, respectively. Therefore, the enhancement due to adding Alumina is more effective than that due to adding Titania because the Alumina-based nanofluid has a smaller relaxation time constant, resulting in a delay in the propagation of the streamer mechanism.

The Electronegativity Theory is the third theory used to explain the enhancement in the impulse breakdown strength of nanofluids. This theory attributes the improvement to the ability of nanoparticles to attract and trap charge carriers due to their high surface potential and electron affinity. In transformer oil contaminated with cellulose particles, interfacial regions are formed between the cellulose and the oil, facilitating local field distortion and streamer initiation. The introduction of nanoparticles modifies this behavior; their electronegative surfaces attract and immobilize free charge carriers, thereby reducing the local charge density at the cellulose-oil interfaces. As a result, the tendency for streamer propagation and pre-breakdown discharges decreases. This mechanism not only suppresses the effect of charges accumulated on cellulose particles but also contributes to a more uniform electric field distribution throughout the oil. Consequently, the nanofluid exhibits a higher impulse breakdown voltage and improved insulation ability.

## 5. Conclusions

This study comprehensively investigated the impact of cellulose contamination on the dielectric performance of transformer oil and explored the use of nanofluids containing titanium dioxide (TiO_2_) and aluminum oxide (Al_2_O_3_) nanoparticles as a remediation strategy. The results revealed that cellulose contamination, even at low concentrations, significantly deteriorates the impulse breakdown strength and polarization capability of insulating oil, primarily by forming conductive bridges and enhancing streamer propagation. Specifically, the addition of 0.02 g/L of cellulose reduced the impulse breakdown voltage of mineral oil by 29.3% and 24.4% under positive and negative polarities, respectively, and decreased the relative permittivity from 2.045 to 2.028. These findings confirm that cellulose particles originating from insulation aging play a destructive role in transformer dielectric systems.

Introducing metal oxide nanoparticles into the contaminated oil effectively reduced these detrimental effects and, in several cases, enhanced the dielectric performance beyond that of pure oil. When 0.02 g/L of TiO_2_ is added to cellulose-contaminated oil, the impulse breakdown voltage increased by 14.6% and 24.4% for positive and negative polarities, respectively, restoring and surpassing the baseline dielectric strength. Similarly, Al_2_O_3_ nanoparticles at the same concentration yielded even greater enhancement, 21.9% and 26.8% for the two polarities, demonstrating that conductive nanoparticles provide stronger electron scavenging and polarization effects than semiconductive ones. Increasing nanoparticle loading to 0.04 g/L produced little further improvement, indicating a saturation limit where excessive concentrations offer no additional benefit due to possible agglomeration.

The relative permittivity and dissipation factor measurements supported the impulse test results, showing that both TiO_2_ and Al_2_O_3_ nanoparticles restored or improved the polarization ability of cellulose-contaminated oil. The addition of Al_2_O_3_ and TiO_2_ yielded the highest dielectric recovery, with ε_r_ rising to 2.048, even greater than 2.045 of standard pure oil. In addition, the dissipation factor roughly becomes equal to that of standard pure oil, to be around 0.002 after reaching 0.0045 when adding cellulose particles as a contaminant, as shown in Figure 18. The observed enhancements can be attributed to three main mechanisms: (i) the moisture-binding capability of hydrophilic nanoparticles, which capture moisture carried by cellulose particles and suppress streamer initiation, (ii) the electron-scavenging process, where nanoparticles trap high-energy electrons, delaying streamer development and strengthening the dielectric medium. The shorter relaxation time constant calculated for Alumina-based nanofluids (41.3 s) compared to Titania-based nanofluids (438.9 s) further explains the superior performance of Al_2_O_3_ in impulse breakdown tests, and (iii) the Electronegativity Theory, which describes the ability of added nanoparticles to attract and trap charge carriers because of their high surface potential.

Overall, the findings demonstrate that nanoparticle-modified transformer oils represent a promising approach to counteract cellulose-induced deterioration in aged transformers. By enhancing dielectric strength and dissipation factor, these nanofluids can extend the service life of insulating systems and improve transformer reliability.

## Figures and Tables

**Table 1:** Specifications of transformer oil used.

Property	IEC 60296 Requirements	Specification
Density at 20 °C (kg/m^3^)	Max. 895	875
Water content (ppm)	Max. 40	25.7
Kinematic viscosity at 40 °C (mm^2^/s)	Max. 12	9.4
Flashpoint (°C)	Min. 135	140
Pour point (°C)	Max. −40	−57
Appearance	Clear	Complies
Total acidity (mg KOH/g)	Max. 1.2	0.9
Dielectric dissipation factor at 90 °C	Max. 0.005	0.002

**Table 2:** Specifications of the Cellulose powder used.

Property	Specification
Form	Microcrystalline Powder
Size	20 μm
pH	5–7 (11 wt.%)
Bulk density	0.5 g/mL (25 °C)

**Table 3:** Specifications of the Nanoparticles used.

Property	Al_2_O_3_	TiO_2_
Form	Nano powder	Nano powder
Particle size	<50 nm	21 nm
Molecular weight	101.96 g/mol	79.87 g/mol
Relative density	4.00 g/cm^3^	4.26 g/cm^3^
Surface area	>40 m^2^/g	35–65 m^2^/g
Melting point	2040 °C	1850 °C

**Table 4:** Parameters of chemicals used.

	$\varepsilon_{r}$	ε	σ
Al_2_O_3_	9.9	87.656 × 10^−12^	1 × 10^−12^
TiO_2_	100	885.42 × 10^−12^	0.1 × 10^−12^
Pure oil	2.045	18.11 × 10^−12^	1 × 10^−12^

## Data Availability

Dataset is available on request from the authors.

## Abbreviations

The following abbreviations are used in this manuscript:

LIBV	Lightning impulse breakdown voltage
BDV	Breakdown voltage
MWS	Maxwell–Wagner–Sillars
TiO_2_	Titanium dioxide
Al_2_O_3_	Aluminum oxide
pH	Potential of hydrogen
IEC	International Electrotechnical Commission
DLS	Dynamic Light Scattering
